# Fabrication of Non-Wetting Mg(OH)_2_ Composites with Photoresponsive Capabilities and Their Environmental Restoration Performance

**DOI:** 10.3390/nano14151240

**Published:** 2024-07-24

**Authors:** Dongmei Zhang, Jiaqi Zhao, Yangyang Peng, Yuchao Li, Wenbin Guo, Chengzhu Liao

**Affiliations:** 1 School of Mathematical Sciences, Liaocheng University, Liaocheng 252059, China; zhangdongmei@lcu.edu.cn (D.Z.); guowenbin@lcu.edu.cn (W.G.); 2 School of Materials Science and Engineering, Liaocheng University, Liaocheng 252059, China; zhaojiaqi200112@163.com (J.Z.); pengyangyanglcu@163.com (Y.P.); 3 Department of Materials Science, South University of Science and Technology, Shenzhen 518055, China

**Keywords:** photodegradation, superhydrophobic, oil–water separation

## Abstract

Water pollution seriously affects the development of society and human life. There are various kinds of pollutants, including soluble pollutants and insoluble floaters on the water surface. Herein, the photocatalyst semiconductor BiOCl and superhydrophobic functional particles Mg(OH)_2_ were deposited on the surfaces of canvas and polyester felt to construct superhydrophobic canvas and polyester felt. The contact angles of the synthetic superhydrophobic canvas and polyester felt were measured as 152° and 155.3°, respectively. The selective adsorption of hexadecane was achieved using the wetting difference between the surface of water and pollutants floating on the surface. For dissolved pollutants, the surface wettability needed to be changed with the help of ethanol. The degradation efficiencies were all greater than 90%, demonstrating the versatility of the synthetic superhydrophobic canvas and polyester felt.

## 1. Introduction

The development of the chemical industry promotes social progress. Nevertheless, the release of industrial pollutants causes the serious destruction of water sources [1,2,3,4]. The treatment of insoluble, oily pollutants and soluble pollutants has become the focus of attention. According to the characteristics of oil-containing pollutants, the efficient removal of oil pollutants requires a special wettability design of the material [5]. Materials that can be wetted by oil rather than water, known as superhydrophobic/superoleophilic materials, are widely applied for herbicide detection [6], oil–water separation [7,8], antibacterial adhesion [9], pressure sensing [10], chromatic paint [11] and radiation cooling [12]. Guan et al. [13] synthesized a superhydrophobic graphene–PLA aerogel through directional freeze drying, which obtained the ability of crude oil adsorption based on the special longitudinal heat-transfer structure. Through plasma treatment and spraying technology, Zhu et al. [14] built a multi-functional composite material that was superhydrophobic on one side and hydrophilic on the other side to realize the oil–water separation. Huang et al. [15] oxidized a cleaned copper mesh to produce Cu(OH)_2_ on its surface and then prepared a superhydrophobic copper mesh through chemical vapor deposition of heptadecafluorodecyl trimethoxysilane. The oil–water separation efficiency was still greater than 97% after continuous use. He et al. [16] used a melamine sponge as a carrier to successively generate polydopamine and Co-HHTP on its surface and, ultimately, obtained a superhydrophobic melamine sponge with polydimethylsiloxane treatment, which realized the efficient separation of various oil–water mixtures. Overall, superhydrophobic materials cannot remove dissolved pollutants due to their limited non-wetting nature despite their selective adsorption capacity to the floating oil in sewage.

In order to achieve the purpose of oil and water separation and removal of water-soluble pollutants, Liu et al. [17] adopted the method of vacuum-assisted extraction and filtration to fabricate a Ti_3_C_2_ membrane, which not only achieved the oil–water separation performance, but also realized the adsorption of water-soluble dyes. However, the adsorbed dyes were prone to cause secondary contamination. Photodegradation technology utilizes active substances produced by sunlight to degrade soluble pollutants, making it an environmentally protective and pollution-free method, and it is extensively applied in the selective oxidation of amines [18], pollutant degradation [19], CO_2_ reduction [20], and NOx removal [21]. Bismuth-based photocatalysts have attracted interest for their adjustable band structure and environmentally friendly properties [22,23]. The special layered structure of [Bi_2_O_2_]^2+^-[Cl]^−^ enables it to possess a special carrier separation ability [24,25,26]. Waehayee et al. [27] synthesized a BiOCl composite material by regulating the exposed crystal surface with HCl. Due to the existence of an internal electric field, the dye degradation was realized. Chen et al. [28] synthesized the oxygen vacancies/BiOCl/TiO_2_ through a two-step hydrothermal method to achieve the degradation of soluble antibiotics. Asim Farid [29] synthesized a Zn_0.98_Mn_0.02_O/BiOCl heterojunction through the precipitation method to fulfill water-soluble dye degradation. Fu et al. [30] synthesized a AgCl/BiOCl heterojunction using a water bath at 90 °C. The special Z-scheme heterojunction achieved a degradation efficiency of 82% for tetracycline. Li et al. [31] synthesized a double-Z-scheme CdS/Bi_2_O_2_CO_3_/BiOCl heterojunction relying on hydrothermal reaction, and the degradation efficiency for the soluble rhodamine B reached 97% in 90 min. Nakayama et al. [32] synthesized a Z-scheme BiOCl@WS_2_ composite through solvothermal and co-precipitation technologies, which showed an excellent degradation performance of water-soluble dyes. The above studies suggest that photocatalysts exhibit the stable degradation of water-soluble pollutants. Water-soluble and non-water-soluble pollutants require an opposite wettability of composite materials. Still, resolving the wetting contradiction remains challenging.

In this study, a simple codeposition method to load BiOCl and superhydrophobic Mg(OH)_2_ onto canvas and polyester felt was developed. Superhydrophobic canvas and polyester felt exhibited excellent non-wettability, with contact angles reaching 152° and 155.3°, respectively. Importantly, with the aid of ethanol, the superhydrophobic surface presented the ability to degrade water-soluble pollutants under light irradiation. The innovation of this paper is the simultaneous removal of water-soluble and non-water-soluble pollutants. The multifunctional surface design is expected to provide a new solution for sewage treatment.

## 2. Experimental Section

### 2.1. Materials

Polyvinylpyrrolidone (Mw = 10,000), diethylene glycol (GC, >98%), magnesium chloride hexahydrate (98%), bismuth nitrate pentahydrate (AR, 99%), and Rhodamine B (AR, Rh B) were provided by Macklin Chemical Reagent Co., Ltd. (Shanghai, China). Potassium chloride (AR, 99.5%) was purchased from Sinopharm Chemical Reagent Co., Ltd. (Beijing, China). Stearic acid (C16: 30%, C18: 70%) and hexadecyl trimethyl ammonium bromide (99%) were provided through Aladdin Chemical Reagent Co., Ltd. (Shanghai, China). Ammonia solution (25~28 wt%) was provided by Yantai Far East Fine Chemical Co., Ltd. (Yantai, China). The curing agent and PDMS were provided by Dow Corning (Midland, MI, USA).

### 2.2. Preparation of Superhydrophobic Composite

A total of 1.67 g of polyvinylpyrrolidone (PVP) was dissolved in 30 mL diethylene glycol, and solution A was obtained after adding 0.73 g Bi(NO_3_)_3_·5H_2_O. Then, 0.34 g KCl was dissolved in 30 mL diethylene glycol to acquire solution B, which was then poured into solution A and stirred for 1 h. The resulting mixture was further transferred into 100 mL hydrothermal reactor at 180 °C for 10 h to obtain BiOCl. In a 250 mL round-bottom flask, 7 g MgCl_2_·6H_2_O and 0.06 g hexadecyl trimethyl ammonium bromide (CTAB) were added to 75 mL of absolute ethanol. The mixture was heated up to 80 °C and refluxed for 30 min. Finally, with the addition of 30 mL of ammonia (25–28%), the reaction continued for 2.5 h to obtain Mg(OH)_2_. A total of 0.2 g of stearic acid was dissolved in 50 mL of absolute ethanol, followed by adding 0.5 g of the above Mg(OH)_2_ under stirring. The mixture was refluxed at 80 °C for 30 min, and the product was centrifuged, washed with absolute ethanol twice, and dried at 80 °C for 4 h to obtain superhyperhydrophobic Mg(OH)_2_, namely SH-Mg(OH)_2_. A total of 0.15 g PDMS and 0.015 g of curing agent were dissolved in 35 mL ethyl acetate. Then, 0.2 g SH-Mg(OH)_2_ and 0.1 g BiOCl were subsequently added and stirred for 10 min. A piece of canvas/polyester felt was placed in the mixture for 0.5 h and then dried at 120 °C for 20 min. The above steps were repeated 4 times to fabricate superhydrophobic canvas and polyester felt.

### 2.3. Characterization

The morphologies and structures of the Mg(OH)_2_ and BiOCl composites were obtained using FESEM (Zeiss, Oberkochen, Germany), TEM (FEI Tecnai F30, Hillsboro, OR, USA), and XRD (TD-3700, Dandong, China). The composition, roughness, and light absorption capacity of the superhydrophobic substrate were analyzed through XPS (Thermo ESCALAB 250Xi, Waltham, MA, USA), AFM (SPA400, Tokyo, Japan), and DRS (Shimadzu UV3600, Tokyo, Japan), respectively. The degradation capabilities and flat-band potentials were analyzed using a UV–visible spectrophotometer (UV-5100H, Shanghai, China) and electrochemical workstation (Corrtest, CS350M, Wuhan, China). The photodegradation experiments were repeated twice, and the average value was taken as the final data. A xenon lamp (300~780 nm, Beijing China) was used as the light source, having a light intensity of 200 mW/cm^2^. Before light irradiation, the samples should be placed in dark for 30 min with a sample interval of 20 min.

## 3. Results and Discussion

**Figure 1:**
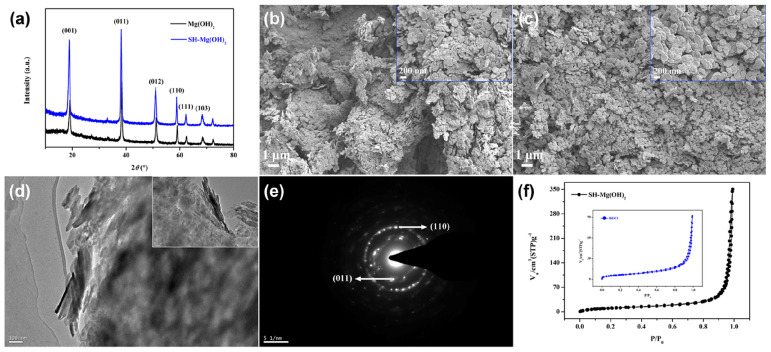
XRD (**a**), FESEM of Mg(OH)_2_ before (**b**) and after (**c**) modification. TEM images of Mg(OH)_2_ before (**d**) and after (inset of **d** and **e**) modification. N_2_ adsorption–desorption isotherms of SH-Mg(OH)_2_ and BiOCl (**f**).

As shown in Figure 1a, the crystal structure of Mg(OH)_2_ after stearic acid modification remained consistent. The crystal planes of (001), (011), (012), (110), (111), and (103) were analyzed. Mg(OH)_2_ before and after stearic acid modification was made up of nanosheets (Figure 1b,c). The morphology observed from TEM images was consistent with FESEM (Figure 1d). The selective electron diffraction image of SH-Mg(OH)_2_ was further analyzed, with crystal planes of (110) and (011) marked (Figure 1e). As shown in Figure 1f, the adsorption isotherms of SH-Mg(OH)_2_ and BiOCl were analyzed as type IV, indicating the mesoporous structure inside the materials. The average pore sizes of SH-Mg(OH)_2_ and BiOCl were 24.6 and 15.2 nm, respectively.

**Figure 2:**
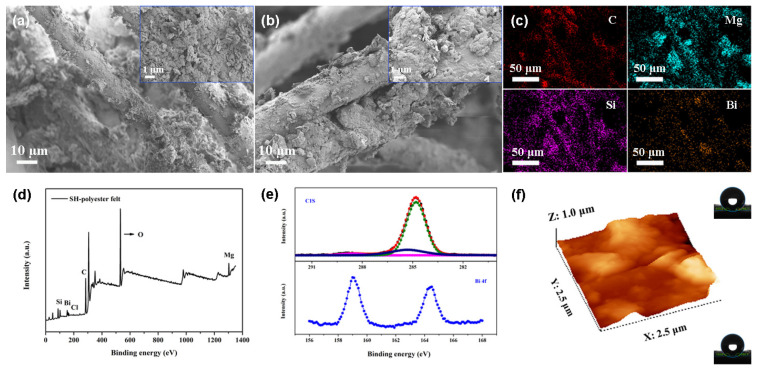
Surface morphology of superhydrophobic canvas (**a**) and polyester felt (**b**). Elemental distribution (**c**), surface composition (**d**,**e**), and roughness (**f**) of superhydrophobic polyester felt.

Functional particles in the powder state are not conducive for reuse. In this paper, the superhydrophobic canvas and polyester felt were successfully synthesized with the help of polydimethylsiloxane. As shown in Figure 2a,b, the superhydrophobic canvas and polyester felt retained the structure of the pristine substrate. The roughness of the loaded particles was increased, which was conducive to achieving non-wetting performance. From the element distribution map of superhydrophobic polyester felt, the distribution trends of the elements C, Mg, Si, and Bi tended to be the same, demonstrating the homogeneity of particles (Figure 2c). As observed, the surface of superhydrophobic polyester felt was composed of Mg, C, Bi, O, Cl, and Si, with contents of 7.6%, 50.5%, 0.3%, 29.8%, 0.4%, and 11.4%, respectively. The higher carbon content helped to reduce the surface tension (Figure 2d). As shown in the XPS fine spectrum of superhydrophobic polyester felt, 159 eV and 164.5 eV were assigned to Bi 4f_7/2_ and 4f_5/2_, respectively. In the spectrum of C1s, the peaks at 285.3 eV, 284.8 eV, and 288.6 eV were attributed to -C-Si, -C-C, and –COO, respectively (Figure 2e). AFM analysis revealed that the roughness of superhydrophobic polyester felt was 117.6 nm (Figure 2f). Both high roughness and low surface tension led to the superhydrophobicity of polyester felt. From the following formula, high roughness is beneficial to reduce the proportion of solid–liquid contact [33]:

(1) $$\cos\theta_{1}=f(\cos\theta_{2}+1)-1$$

where *θ*_1_ and *θ*_2_ represent the contact angle with the Cassie–Baxter state and Young’s contact angle, respectively. *f* is the proportion of solid–liquid contacts. Moreover, the surface contact angles of the superhydrophobic canvas and polyester felt reached 152° and 155.3°, respectively (inset of Figure 2f).

**Figure 3:**
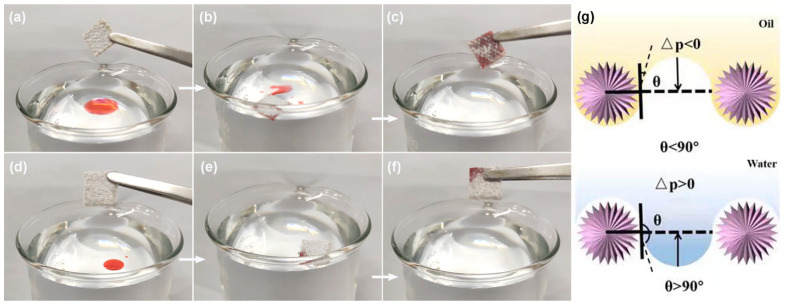
Selective oil removal performance of superhydrophobic canvas (**a**–**c**) and polyester felt (**d**–**f**), of which hexadecane is labeled red. Mechanism of oil–water separation (**g**).

For ease of observation, the non-water-soluble contaminant (hexadecane) was colored in red. When placing the superhydrophobic canvas (Figure 3a–c) and polyester felt (Figure 3d–f) with excellent non-wettability at the pollutant–water interface, hexadecane rapidly expanded on the surface, while water could not wet the solid surface, realizing selective oil removal. For the removal of water-soluble pollutants, both adsorption and photodegradation require wettability. Therefore, a simple ethanol immersion method was employed to change the wettability from superhydrophobicity to superhydrophilicity. The mechanism of the oil–water separation can be explained through the Laplace equation [34]:

(2) $$\Delta p=\frac{2\sigma }{R}=-\frac{l\sigma \cos\theta }{A}$$

where, *σ*, *R*, *l*, and *A* indicate the surface tension, radius of curvature, perimeter, and area of the hole, respectively. When the superhydrophobic fabric and polyester felt contacted the oil–water mixture, the oil phase was subjected to a downward force and was thus adsorbed, whereas the water phase was subjected to an upward force, which was repulsed on the surface to achieve oil–water separation (Figure 3g).

**Figure 4:**
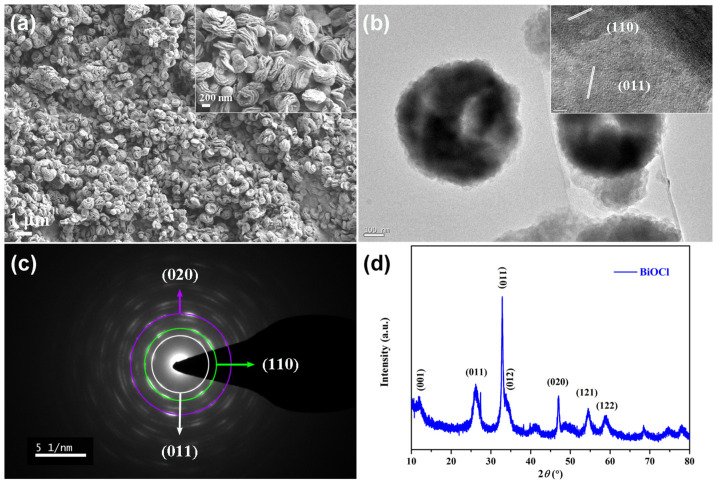
Surface morphology (**a**), TEM image (**b**), SAED image (**c**), and XRD analysis (**d**) of BiOCl.

It should be noted that Rh B was used as the water-soluble contaminant. Due to the presence of BiOCl, the absorbed light energy was converted to active species to degrade Rh B. According to Figure 4a, the synthetic BiOCl displayed a self-assembled floral morphology. TEM images of BiOCl demonstrated the same morphology as FESEM, and the crystal planes of (110) and (011) were analyzed through HRTEM image (Figure 4b). The crystal planes of (020), (110), and (011) were classified (Figure 4c). The crystal planes of (001), (011), (110), (012), (020), (121), and (122) were identified through XRD analysis (Figure 4d). The above analysis validated the successful synthesis of BiOCl.

**Figure 5:**
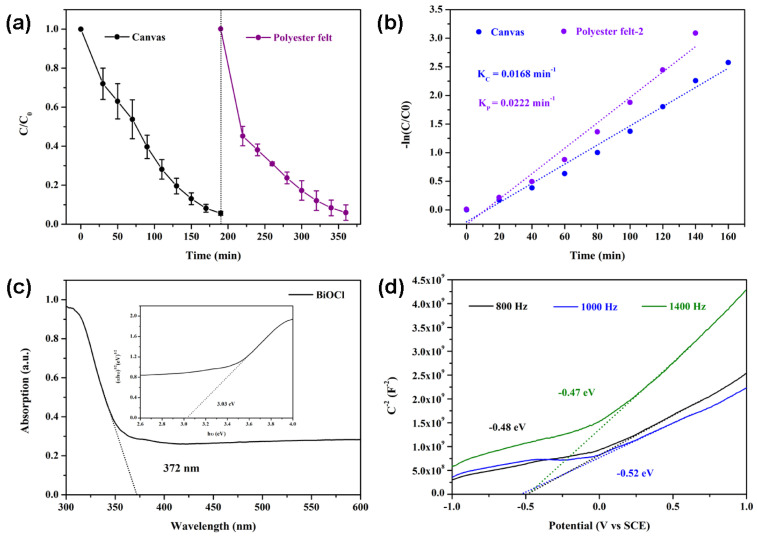
Photodegradation behavior (**a**) and reaction kinetics (**b**) of superhydrophobic canvas and polyester felt. DRS (**c**) and Mott–Schottky curve (**d**) analysis of BiOCl.

Both superhydrophobic canvas and polyester felt exhibited degradation properties to water-soluble Rh B, with degradation efficiencies of 94.4% and 94.2%, respectively (Figure 5a). The photodegradation reaction belongs to the first-order reaction. Therefore, with –ln(C/C_0_) as the ordinate and time as the abscissa, the degradation kinetics of superhydrophobic canvas and polyester felt were 0.0168 min^−1^ and 0.0222 min^−1^, respectively. Photoresponsiveness is an important indicator for the evaluation of photocatalysts. As shown in Figure 5c, the cut-off wavelength of BiOCl was 372 nm. With (αhυ)^1/2^ as the ordinate and hυ as the abscissa, the calculated band width of BiOCl was 3.03 eV. From the Mott–Schottky curve of BiOCl, the synthesized BiOCl belonged to the N-type semiconductor with a flat-band position of −0.49 eV vs. SCE and −0.245 eV vs. NHE (Figure 5d). Analysis of the degradation products revealed that their TOC and COD were 1963 and 12,888 mg/L, respectively.

**Figure 6:**
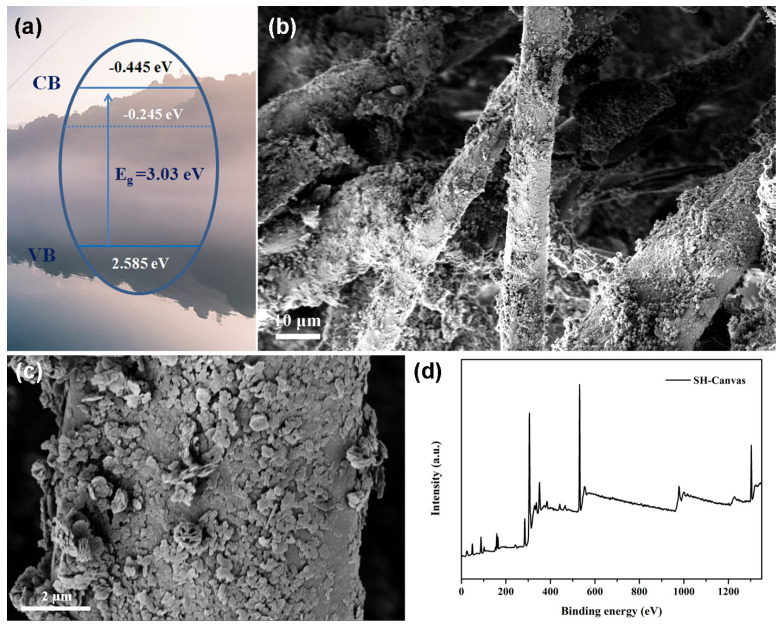
Photodegradation mechanism of BiOCl (**a**). Surface morphology (**b**,**c**) and composition (**d**) analysis of superhydrophobic canvas after degradation experiments.

For N-type semiconductors, the distance between the flat charged site and CB is between 0.1 eV and 0.2 eV [35]. The calculated conduction band position of BiOCl was −0.445 eV, and the valence band position was 2.585 eV. Under light irradiation, electrons in the valence band of BiOCl transitioned to the conduction band, which then formed active species to degrade soluble pollutants (Figure 6a). Additionally, there was no significant change in the surface morphology of the canvas after photodegradation. The woven structure of the canvas, the morphologies of BiOCl, and superhydrophobic Mg(OH)_2_ were clearly observed (Figure 6b,c). After photodegradation, the surface elements of the superhydrophobic canvas were analyzed, whose surface was composed of Bi, O, Cl, C, Mg, and Si, with contents of 0.7%, 39.2%, 0.7%, 27.8%, 24%, and 7.7%, respectively (Figure 6d).

## 4. Conclusions

Non-wetting materials are widely used in oil–water separation, but they cannot degrade water-soluble pollutants. In this paper, superhydrophobic Mg(OH)_2_ and BiOCl were co-precipitated on the surfaces of canvas and polyester felt to construct superhydrophobic canvas and polyester felt, with the surface contact angles to water reaching 152° and 155.3°, respectively. The substrate and loaded functional particles provided the roughness required for superhydrophobic properties, which contributed to realization of the oil–water separation performance. In addition, photodegradation of water-soluble dyes was achieved, aided by ethanol. The reaction kinetics of the superhydrophobic canvas and polyester felt were 0.0168 min^−1^ and 0.0222 min^−1^, respectively. Through Mott–Schottky and DRS analyses, the valence and conduction band positions of BiOCl were measured as 2.585 and −0.445 eV, respectively. Eventually, the superhydrophobic canvas and polyester felt achieved oil–water separation and photodegradation of water-soluble pollutants.

## Data Availability

Data are contained within the article.
